# Androgen receptor and its correlation with estrogen and progesterone receptors, aimed for identification of cases for future anti-androgen therapy in endometrial cancers

**DOI:** 10.1371/journal.pone.0291361

**Published:** 2023-09-19

**Authors:** Neda A. Moatamed, Saba Vahdatshariatpanahi, David W. Gjertson, Chana R. Sachs, Yuna Kang, Nora Ostrzega, Jiaoti Huang, Sanaz Memarzadeh

**Affiliations:** 1 Department of Pathology and Laboratory Medicine, David Geffen School of Medicine at UCLA, Los Angeles, California, United States of America; 2 Department of Biostatistics, Fielding School of Public Health at UCLA, Los Angeles, California, United States America; 3 Department of Pathology, Duke University School of Medicine, Durham, North Carolina, United States of America; 4 Department of Obstetrics and Gynecology, David Geffen School of Medicine at UCLA, Los Angeles, California, United States of America; 5 UCLA Eli and Edythe Broad Center of Regenerative Medicine and Stem Cell Research, University of California Los Angeles, Los Angeles, California, United States America; 6 Johnson Comprehensive Cancer Center, University of California Los Angeles, Los Angeles, California, United States of America; 7 Molecular Biology Institute, University of California Los Angeles, Los Angeles, California, United States of America; 8 VA Greater Los Angeles Healthcare System, Los Angeles, California, United Sates of America; QUT Health: Queensland University of Technology Faculty of Health, AUSTRALIA

## Abstract

**Introduction:**

The expression of androgen receptor (AR) is not commonly tested or studied in uterine cancers, unlike estrogen receptor (ER) and progesterone receptor (PR) which are positive in most endometrial carcinomas. In this series, we evaluated the expression of AR and its comparison to ER and PR in different types of endometrial cancers and have reviewed the literature.

**Materials and methods:**

The status of AR, ER, and PR expression were evaluated in 71 cases which were categorized into endometrial endometrioid cancer (EEC), non-endometrioid endometrial cancers (NEEC), and metastatic carcinomas of endometrium. Expression of the receptors were compared to each other as well as to mismatch repair proteins (MMR), p53, and body mass index (BMI) using Fisher’s Exact test in the StatPlus software.

**Results:**

In EECs, the positivity was 97% for all the three receptors. In NEEC, positivity rates were 68%, 48%, and 35% for AR, ER, and PR respectively. In Metastatic carcinomas, AR and ER positivity was seen in 100% while PR was positive in 75% of the cases. In all cancers, the rates were 17% (11/66) for MMR loss, 57% (30/53) for p53 aberrant expression, and 76% (54/71) for the patients with BMI of ≥ 25 (kg/m2).

**Conclusion:**

AR is expressed in a high percentage of endometrial cancers. Its significance is more evident in high-grade NEEC where ER and PR may not be expressed. These findings warrant further evaluation of AR expression and candidacy of this pathway as a potential therapeutic target in endometrial cancers.

## Introduction

Endometrial cancers (ECs) are the most common gynecologic malignancies in the United States [1]. The growth of these cancers may be regulated by some hormones, particularly estrogen, which promotes proliferation of most ECs [2]. There are different histologic subtypes of ECs which include endometrioid, serous, clear cell, mixed types, undifferentiated/dedifferentiated, mesonephric-like adenocarcinoma, and carcinosarcoma [3]. Traditionally, endometrial cancers have been categorized into two types. In type-1, they are estrogen derived low grade endometrioid carcinomas and type-2s are generally high-grade cancers which are not estrogen derived [4]. Recent studies have suggested that serous carcinomas may also be associated with hormone exposure [5]. Progesterone can stop estrogen driven proliferation and can be used in the therapy for low grade endometrioid carcinomas [2]. Some of the non-gynecological neoplasms, such as prostate cancer which express androgen receptor (AR), have been treated with androgen antagonist for many years [6]. Also, AR-targeted treatment for triple negative breast cancers, including apocrine type breast carcinoma, has been an area of active investigation [7–9].

The effect of androgen action in endometrial cancer is poorly understood. ARs are expressed in the endometrium throughout the menstrual cycle, even as androgens play a role in the regulation of endometrial apoptosis [10]. While in theory, androgen has an antiproliferative effect in the normal endometrium, there are studies which suggest that androgen may be important in the growth of EC and ovarian cancer [4, 11]. Adipose tissue is the primary site of estrogen production in post-menopausal women coincident with increased rate of ECs in obese females [11]. Elevated circulating levels of free testosterone have been seen in females with EC [12]. Also, free testosterone and dehydroepiandrosterone have been reported to correlate with abdominal fatty tissue in post-menopausal women [13]. Experiments have shown that enzalutamide, an anti-androgen drug may have a growth inhibitory effect in endometrial cancer cells [14]. While hormonal therapy targeting ER and PR is used in treatment of patients with endometrial cancers [15], there are limited studies evaluating the effects of hormone therapy targeting AR [14]; complicated by the contradictory findings regarding the androgen levels in females, as mentioned above.

The purpose of this study was to investigate the expression of AR and compare the findings to that in ER and PR among the different types of ECs.

## Materials and methods

This retrospective study was approved by the Institutional Review Board (IRB #22–000672) at UCLA David Geffen School of Medicine. Since this project was a retrospective study on the archived information and the tissue blocks, no patients were involved and the need for patient’s consent had been waived by the UCLA IRB. The Epic Beaker laboratory information system was used to conduct a search for endometrial cancers between March 2016 to June 2022. The inclusion criteria for the search included cases who had ECs, had been tested for estrogen receptor (ER) and progesterone receptor (PR) by immunohistochemical (IHC) stains for patient care and their results had been reported. Among which, mismatch repair (MMR) and p53 test results were also obtained if they had been ordered during the routine pathology practice. In addition, some demographic information, including age, body mass index (BMI), surviving months following the initial diagnosis, and the treatments, was recorded. For all primary endometrial malignancies, the nomenclature and FIGO (International Federation of Gynecology and Obstetrics) grades were recorded according to the established WHO guidelines [3].

### IHC stains

Leica BOND III auto-Stainer was used to carry out the staining steps. Rabbit monoclonal anti-AR antibody (clone: SP107) was obtained from Cell Marque and used at 1:100 dilution. The paraffin sections were deparaffinized and the epitopes were retrieved in BOND-ER-2 solution for 30 minutes followed by the antibody incubation for an additional 15 minutes. BOND Refine Detection kit was used to detect the bonded antibody molecules.

The IHCs for ER, PR, mismatch repair (MMR), and p53 had already been performed for the clinical or diagnostic purposes on these cases and the data were available in the patients’ clinical records. Only AR IHC was carried out on the respective tissue sections for this study.

For ER IHC, Epredia rabbit monoclonal anti-ER (clone SP1) was used. Additionally, anti-PR monoclonal mouse antibody (clone PgR-636) had been obtained from DAKO. ER and PR IHC staining was carried out according to the established methodology in our department which was similar to AR as described. Breast tissues were used as control for ER and PR, while prostate and testicular tissues were utilized as control for AR.

For MMR IHC stains, MLH1 (mouse monoclonal antibody, clone G168-728) and MSH2 (mouse monoclonal antibody, clone G219-1129) had been obtained from Cell Marque. PMS2 (rabbit monoclonal antibody, clone EP51) and MSH6 (rabbit monoclonal antibody, clone EP49) had been utilized from Epitomics and using normal cellular components as control.

For p53 IHC, mouse monoclonal antibody (clone DO-7) by Novacastra, had been obtained from Leica using high-grade serous carcinoma tissue as control.

### IHC stain scoring

Scoring for ER and PR had been carried out as per the current American Society of Clinical Oncology (ASCO)/College of American Pathologists (CAP) guidelines for breast cancer where any cellular reaction of 1% or more is considered as positive [16, 17]. An identical procedure was adopted for the AR IHC scoring for this study. Weak nuclear staining was recorded as 1+, moderate nuclear staining as 2+, and strong nuclear expression as 3+ intensities. Both the percentage of positive cancer cells and the intensity of the stain were recorded for future meta-analysis. All the stains were performed on whole tissue sections. No tissue micro-array was used. The stains were re-reviewed by two of the authors and the discrepant cases were additionally double scoped by two other pathologists. The final stain interpretations were recorded for this series.

### Mismatch repair (MMR)

Loss of MMR protein expression is widely tested by IHC throughout the world [18]. The stains were targeted for MLH1, PMS2, MSH2, and MSH6 genes. Any loss of the respective protein was marked as positive. Lack of the tests resulted in exclusion of the cases for MMR evaluation. The intact proteins were labeled as negative for the statistical calculations.

### p53

TP53 missense mutation tends to accumulate p53 protein in the nuclei of the cancer cells. Normally, the scattered nuclear IHC reaction which is present in less than 30% of the cells, is referred to as wild type (WT). There are two general IHC patterns when the mutation occurs, one is complete absence of the reaction and second is diffuse and strong nuclear and sometimes cytoplasmic staining of more than 80% of the cells. The two patterns are referred to as aberrant expressions (AE) [19]. The AEs were referred to as positive and WT as negative for the statistical analyses in this series. Lack of the test performance led to exclude cases for p53 evaluation.

### Body mass index (BMI)

We used body mass indices (kg/m^2^) as defined in WHO guidelines: underweight (BMI < 18.5), normal or desirable weight (BMI = 18.5 to <25), over-weight (BMI = ≥25 to <30), and three classes of obesity (BMI ≥30) [20]. In this study, BMIs of <25 (normal + underweight) as negative and BMIs of ≥25 (Overweight + obese) as positive were used to compare to the receptors for concordance.

### Statistical analysis

Fisher’s Exact test, in a 2x2 contingency table [21], was used to compare the AR, ER, and PR IHC stains to each other as well as comparing the receptors’ stains to MMR, p53, and BMI. To reject null hypothesis, *p*-values of 0.05 or less were used to indicate a significant difference between two data sets.

### Study design

Androgen, estrogen, and progesterone receptors’ stain results were tabulated based on three diagnostic categories: EEC (endometrial endometrioid cancer), NEEC (non-endometrioid endometrial cancer), and MET (metastatic carcinoma). The NEEC category included high-grade malignancies comprised of serous carcinoma (SCA), clear cell carcinoma (CCCA), malignant mixed Müllerian tumor or carcinosarcoma (CS), dedifferentiated/undifferentiated endometrial carcinoma (DECA), and mesonephric-like adenocarcinoma (MNCA). The three steroid hormone receptors were considered positive when the nuclear stains were present in 1% or more of the cancer cells [16, 17]. The estimated percentage of the cellular positivity and the intensity of the stains were all tabulated (S1 Table). Fisher’s Exact test was carried out in 2x2 contingency tables using negative and positive results in the three diagnostic categories comparing the three receptor stains to each other as well as to MMR, p53, and BMI.

In the design and conduct of this work, we adhered to the parts of REMARK guidelines germane to our study [22]. Since we had not used androgen antagonists or anti-AR agents on the patients in a clinical trial, the survival or outcome aspect of the guidelines was not applicable to the current series [22].

## Results

Among the 937 cases with endometrial cancers obtained during the search period, the computer returned 71 surgical pathology cases matching the inclusion criteria. For this study, AR stain was performed and evaluated on these 71 cases. Patients’ ages ranged from 41 to 88 years with a median of 65. All patients were postmenopausal except for three (cases # 20, 33, and 50; S1 Table). Thirty-seven cases, mostly with high-grade cancers, had chemotherapy among which two had received additional anti-estrogen (anastrozole) treatment (cases # 26 same as 71 and 68, S1 Table). None of the cases had received treatments with anti-androgen agents. Twelve patients had expired at the conclusion of this study on May 13, 2023 (S1 Table).

The cancer cell positivity ranged from 3% to 95% for all the three receptors’ immuno-stains (S1 Table). Overall, ER was positive in 76% (54/71), PR was positive in 69% (49/71), and AR was positive in 85% (60/71) of all malignancies (“All” category) of which combined 2+ & 3+ intensities were observed in 65% (46/71) of ER & PR, and 82% (58/71) of AR reactions as listed in Table 1 and S1 Table. These findings indicate higher rates of positivity and stronger intensity (expression) of AR.

**Table 1:** Summary of data including the results of the immunostains and Fisher’s Exact tests.

Diagnosis	n	ER		PR		AR	
		+n	+%	+n	+%	+n	+%
**EEC**	**36**	**35**	**97%**	**35**	**97%**	**35**	**97%**
**NEEC**	**31**	**15**	**48%**	**11**	**35%**	**21**	**68%**
SCA	13	11	85%	8	62%	12	92%
CCCA	6	0	0%	0	0%	3	50%
CS	6	4	67%	3	50%	6	100%
DECA	5	0	0%	0	0%	0	0%
MNCA	1	0	0%	0	0%	0	0%
**Met**	**4**	**4**	**100%**	**3**	**75%**	**4**	**100%**
**All**	**71**	**54**	**76%**	**49**	**69%**	**60**	**85%**
**2x2 Contingency Table Values**				**Fisher’s Exact Test *P*-Values**			
		**Positive**	**Negative**	**ER** vs **PR**	**AR** vs **ER**	**AR** vs **PR**	
**EEC**	**ER**	35	1			1	
	**PR**	35	1		1		
	**AR**	35	1	1			
**NEEC**	**ER**	15	16			**0.02**	
	**PR**	11	20		0.20		
	**AR**	21	10	0.4			
**Met**	**ER**	4	0			1.0	
	**PR**	3	1		1*		
	**AR**	4	0	1.0			
**All**	**ER**	54	17			**0.05**	
	**PR**	49	22		0.29		
	**AR**	60	11	0.5			

**ER**, estrogen receptor; **PR**, progesterone receptor; **AR**, androgen receptor; **+**, positive reactions; **EEC**, endometrial endometrioid cancer; **NEEC**, non-endometrioid endometrial cancers which include: **SCA**, serous carcinoma; **CCCA**, clear cell carcinoma; **CS**, carcinosarcoma; **DECA**, dedifferentiated endometrial carcinoma; and **MNCA**, mesonephric like carcinoma; **Met**, metastatic carcinoma; **1**

*, although the statistical test did net return any result because of the two zeros, the *p*-value is assumed as "1" since both sets of data are identical.

When the three stains were compared against each other in combined three diagnostic categories (EEC, NEEC, and Met), Fisher Exact tests did not return significant *p*-values except for AR versus PR (*p*-value = 0.05) where 60 cases were positive for AR as opposed to 49 for PR (Table 1).

There were 66 cases (93% of the total) who had MMR IHC tests performed for patient care purposes. Mismatch repair gene proteins had been lost in 17% (11/66) of cases and were intact in 83% (55/66) of the patients (S1 Table). Cases with no MMR tests were excluded from the statistical analysis. Fisher’s Exact test showed significant differences (*p*-values of <0.0001) indicating non-concordance of MMR with 17% (11/66) losses as to 76% (50/66) for ER, 68% (45/66) for PR, and 85% (56/66) for AR expression (Table 2).

**Table 2:** Summary of data including the results of the receptors and MMR immunostains, and Fisher’s Exact tests.

Diagnosis	n	ER		PR		AR		MMR	
		+n	+%	+n	+%	+n	+%	+n	+%
**EEC**	**36**	**35**	**97%**	**35**	**97%**	**35**	**97%**	**6**	**17%**
**NEEC**	**26**	**11**	**42%**	**7**	**27%**	**17**	**65%**	**3**	**12%**
SCA	10	8	80%	5	50%	9	90%	0	**0%**
CCCA	5	0	0%	0	0%	3	60%	0	**0%**
CS	5	3	60%	2	40%	5	100%	0	**0%**
DECA	5	0	0%	0	0%	0	0%	3	**60%**
MNCA	1	0	0%	0	0%	0	0%	0	**0%**
**Met**	**4**	**4**	**100%**	**3**	**75%**	**4**	**100%**	**2**	**50%**
**All**	**66**	**50**	**76%**	**45**	**68%**	**56**	**85%**	**11**	**17%**
**2x2 Contingency Table Values**					**Fisher’s Exact Test *P*-Values**				
		**Positive**	**Negative**	**ER** vs **MMR**		**PR** vs **MMR**		**AR** vs **MMR**	
**EEC**	**ER**	35	1			**<0.0001**		** **	
	**PR**	35	1	**<0.0001**		** **		**<0.0001**	
	**AR**	35	1	** **					
	**MMR**	6	30	** **					
**NEEC**	**ER**	11	15			0.3		** **	
	**PR**	7	19	**0.03**		** **		**0.0001**	
	**AR**	17	9	** **					
	**MMR**	3	23	** **					
**Met**	**ER**	4	0			1		** **	
	**PR**	3	1	0.4		** **		0.4	
	**AR**	4	0	** **					
	**MMR**	2	2	** **					
**All**	**ER**	50	16			**<0.0001**		** **	
	**PR**	45	21	**<0.0001**		** **		**<0.0001**	
	**AR**	56	10	** **					
	**MMR**	11	55	** **					

**ER**, estrogen receptor; **PR**, progesterone receptor; **+**, positive reaction; **AR**, androgen receptor; **EEC**, endometrial endometrioid cancer; **NEEC**, non-endometrioid endometrial cancers which includes: **SCA**, serous carcinoma; **CCCA**, clear cell carcinoma; **CS**, carcinosarcoma; **DECA**, dedifferentiated endometrial carcinoma; and **MNCA**, mesonephric like carcinoma; **Met**, metastatic carcinoma; **MMR**, mismatch repair, loss as positive, intact as negative.

Among the 71 cases, 53 (75%) had p53 IHC tests performed for patient care purposes. The cases with no p53 were excluded from the statistical test. There were 57% (30/53) of patients who had aberrant expression of p53 (S1 Table). The receptors’ IHC stain results were individually compared to p53 in all 53 patients. Fisher’s Exact test showed a significant difference when p53 was compared to AR (*p*-value = 0.01), where p53 had an aberrant expression in 57% (30/53) whereas AR had been expressed in 81% (43/53) of the cases. The comparative differences between p53 and ER/PR were not statistically significant (Table 3).

**Table 3:** Summary of data including the results of the receptors and p53 immunostains, and Fisher’s Exact tests.

**Diagnosis**	**n**	**ER**		**PR**		**AR**		**p53**	
		+n	+%	+n	+%	+n	+%	+n	+%
**EEC**	**24**	**23**	**96%**	**23**	**96%**	**23**	**96%**	**3**	**13%**
**NEEC**	**28**	**13**	**46%**	**10**	**36%**	**19**	**68%**	**27**	**96%**
SCA	13	11	85%	8	62%	12	92%	13	100%
CCCA	5	0	0%	0	0%	3	60%	5	100%
CS	4	2	50%	2	50%	4	100%	4	100%
DECA	5	0	0%	0	0%	0	0%	5	100%
MNCA	1	0	0%	0	0%	0	0%	0	0%
**Met**	**1**	**1**	**100%**	**0**	**0%**	**1**	**100%**	**0**	**0%**
**All**	**53**	**37**	**70%**	**33**	**62%**	**43**	**81%**	**30**	**57%**
**2x2 Contingency Table Values**					**Fisher’s Exact Test *P*-Values**				
		**Positive**	**Negative**	**ER** vs **p53**		**PR** vs **p53**		**AR** vs **p53**	
**EEC**	**ER**	23	1			**<0.0001**			
	**PR**	23	1	**<0.0001**				**<0.0001**	
	**AR**	23	1						
	**p53**	3	21						
**NEEC**	**ER**	13	15			**<0.0001**			
	**PR**	10	18	**<0.0001**				**0.01**	
	**AR**	19	9						
	**p53**	27	1						
**Met**	**ER**	1	0			1*			
	**PR**	0	1	1				1	
	**AR**	1	0						
	**p53**	0	1						
**All**	**ER**	37	16			0.7			
	**PR**	33	20	0.2				**0.01**	
	**AR**	43	10						
	**p53**	30	23						

**ER**, estrogen receptor; **PR**, progesterone receptor; **AR**, androgen receptor; **+**, positive reactions; **EEC**, endometrial endometrioid cancer, **NEEC**, non-endometrioid endometrial cancers which include: **SCA**, serous carcinoma; **CCCA**, clear cell carcinoma; **CS**, carcinosarcoma; **DECA**, dedifferentiated endometrial carcinoma; and **MNCA**, mesonephric like carcinoma; **Met**, metastatic carcinoma; **p53**, aberrant-expression as positive, wild-type as negative

*, although the statistical test did net return any result because of the two zeros, the *p*-value is assumed as "1" since both sets of data are identical.

BMIs of 25 (kg/m^2^) or greater were observed in 76% (54/71) of the total patients, among which 48% (34/71) had a BMI of 30 or more falling in the obese classes [20]. Fisher’s Exact test did not return significant *p*-values when the receptor stains were compared to BMIs of ≥25 in the “All” category, indicating concordance of the BMIs with receptor stains (Table 4).

**Table 4:** Summary of the data including the receptors immunostain results and Fisher’s Exact tests’ *p*-values as the stains have been compared to the body mass index.

Diagnosis	n	ER		PR		AR		BMI	
		+n	+%	+n	+%	+n	+%	+n	+%
**EEC**	**36**	**35**	**97%**	**35**	**97%**	**35**	**97%**	**25**	**69%**
**NEEC**	**31**	**15**	**48%**	**11**	**35%**	**21**	**68%**	**25**	**81%**
SCA	13	11	85%	8	62%	12	92%	12	92%
CCCA	6	0	0%	0	0%	3	50%	5	83%
CS	6	4	67%	3	50%	6	100%	4	67%
DECA	5	0	0%	0	0%	0	0%	4	80%
MNCA	1	0	0%	0	0%	0	0%	0	0%
**Met**	**4**	**4**	**100%**	**3**	**75%**	**4**	**100%**	**4**	**100%**
**All**	**71**	**54**	**76%**	**49**	**69%**	**60**	**85%**	**54**	**76%**
**2x2 Contingency Table Values**					**Fisher’s Exact Test *P*-Values**				
		**Positive**	**Negative**	**ER** vs **BMI**		**PR** vs **BMI**		**AR** vs **BMI**	
**EEC**	**ER**	35	1			**0.003**		** **	
	**PR**	35	1	**0.003**		** **		**0.003**	
	**AR**	35	1	** **					
	**BMI**	25	11	** **					
**NEEC**	**ER**	15	16			**0.0007**		** **	
	**PR**	11	20	**0.02**		** **		0.4	
	**AR**	21	10	** **					
	**BMI**	25	6	** **					
**Met**	**ER**	4	0			1		** **	
	**PR**	3	1	1*		** **		1*	
	**AR**	4	0	** **					
	**BMI**	4	0	** **					
**All**	**ER**	54	17			0.5		** **	
	**PR**	49	22	1		** **		0.3	
	**AR**	60	11	** **					
	**BMI**	54	17	** **					

**ER**, estrogen receptor; **PR**, progesterone receptor; **AR**, androgen receptor; **+**, positive reaction; **EEC**, endometrial endometrioid cancer; **NEEC**, non-endometrioid endometrial cancers which includes: **SCA**, serous carcinoma; **CCCA**, clear cell carcinoma; **CS**, carcinosarcoma; **DECA**, dedifferentiated endometrial carcinoma; and **MNCA**, mesonephric like carcinoma; **Met**, metastatic carcinoma; **BMI**, body mass index (kg/m2), BMI ≥30 as positive, BMI <30 as negative

*, the statistical test did net return any result because of the two zeros, therefor the *p*-value is assumed as "1" since both sets of data are identical.

Due to the discovery nature of this study, *p*-values were not corrected for multiple comparisons, and their nominal values should be interpreted cautiously.

### EEC (endometrial endometrioid cancer)

There were 36 cases in this diagnostic category. Patients’ ages ranged between 41 to 88 with a median of 65 years. All patients were postmenopausal except for two (cases # 20 and 33; S1 Table). Six patients had chemotherapy among which one had received additional anti-estrogen (anastrozole) treatment. None of the patients had been treated with anti-androgen agents. Two patients had expired when this study ended on May 13, 2023 (S1 Table).

**Fig 1:**
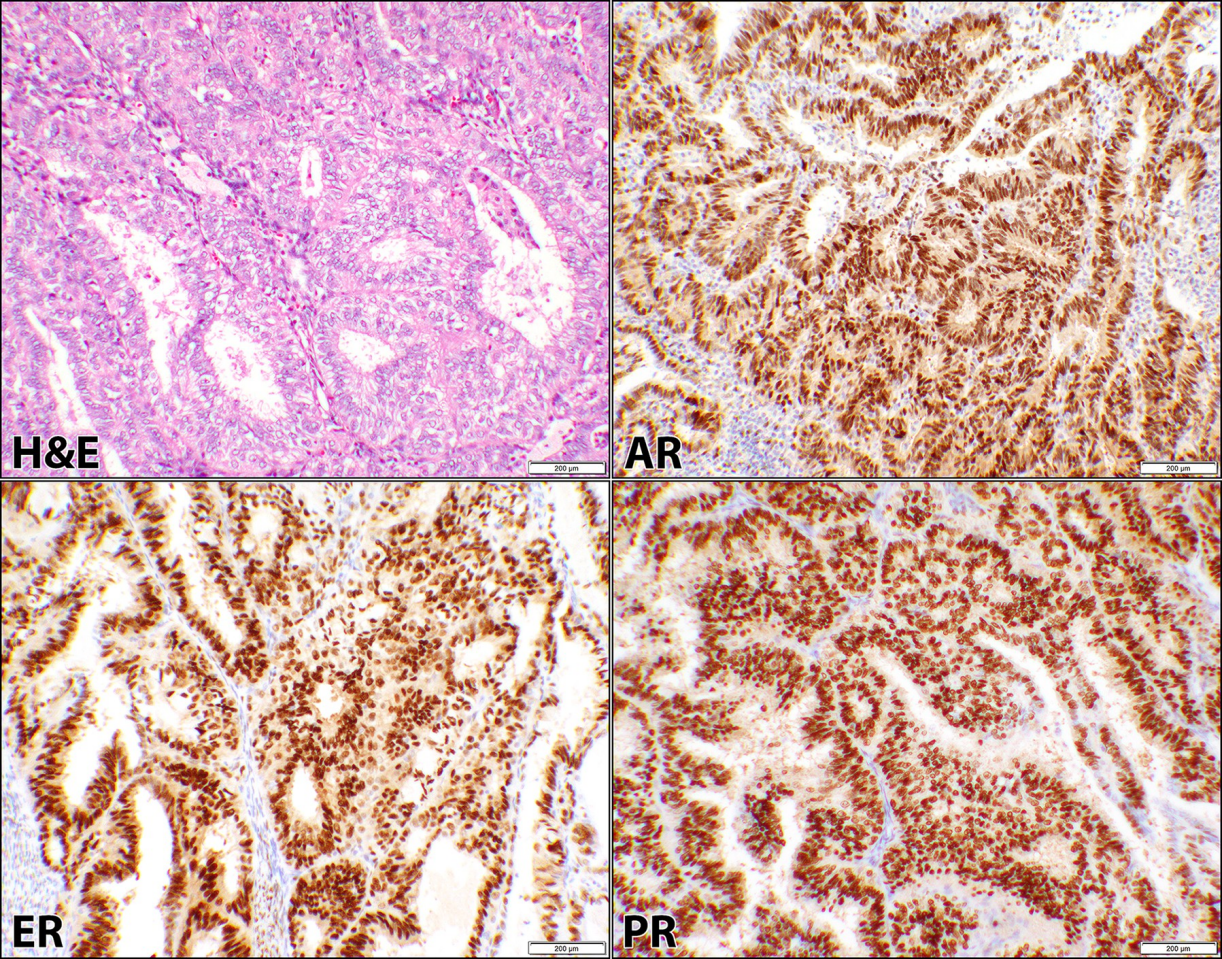
Androgen, estrogen, and progesterone receptors in endometrioid carcinoma. Hematoxylin and eosin (H&E) stain of a case (case #30, S1 Table) of uterine endometrioid carcinoma showing glandular proliferation, composed of columnar cells with pseudostratified nuclei and mild to moderate atypia. The corresponding androgen receptor (AR), estrogen receptor (ER), and progesterone receptor (PR) immunostains show similar positive nuclear staining of the malignant cells (10× objective).

Twenty cases were FIGO grade-1, thirteen had grade-2, and three with grade-3. AR, ER, and PR stains were positive in 97% (35/36) of the patients as shown in Table 1 and represented in Fig 1. Combined 2+ & 3+ intensities of the respective reactions were observed in 86% (31/36), 92% (33/36), and 94% (34/36) of ER, PR, and AR respectively. Only one low grade EEC (case #11, S1 Table) was negative for the IHC reactions. Overall, the cellular positivity ranged from 5% to 90% with the intensity of 1+ to 3+ for all three receptors (S1 Table). Since the positivity of the cells were similar for the three receptors, Fisher’s Exact test had returned a *p*-value of “1” when the stains were compared to each other in this diagnostic category (Table 1).

All specimens, in this category, had been tested for MMR in which the stains showed 83% (30/36) intact proteins whereas loss of MLH1 and PMS2 were seen in 4 and loss of MSH2 and MSH6 were observed in 2 cases (S1 Table). MMR loss was significantly different when compared to each receptor using Fisher’s Exact test (*p*-values of <0.0001) indicating lack of concordance with 17% MMR loss versus 97% ER/PR/AR expressions (Table 2). Other comparisons involved p53 and BMI. p53 was also significantly different from the three receptors (*p*-values of <0.0001), again non-concordant with 13% versus 96% of the three receptors (Table 3).

Among the 36 patients, 69% (25/36) were overweight (MBI ≥ 25) of which 44% (16/36) were obese (BMI ≥ 30) in this category. Comparison of the BMI to the receptors, resulted in significant differences (*p*-values of 0.003) by Fisher’s Exact test where 69% of the patients were overweight (BMI ≥ 25) as opposed to 97% ER, PR, and AR expressions (Table 4).

### NEEC (non-endometrioid endometrial cancers)

There were 31 cases in this diagnostic category. Patients’ ages ranged between 45 to 83 with a median of 68 years. All patients were postmenopausal except for one (cases # 50; S1 Table). Twenty-seven patients had chemotherapy among which none had received additional anti-estrogen (anastrozole) treatment or anti-androgen agents. Ten patients had expired when this study was concluded on May 13, 2023 (S1 Table).

**Fig 2:**
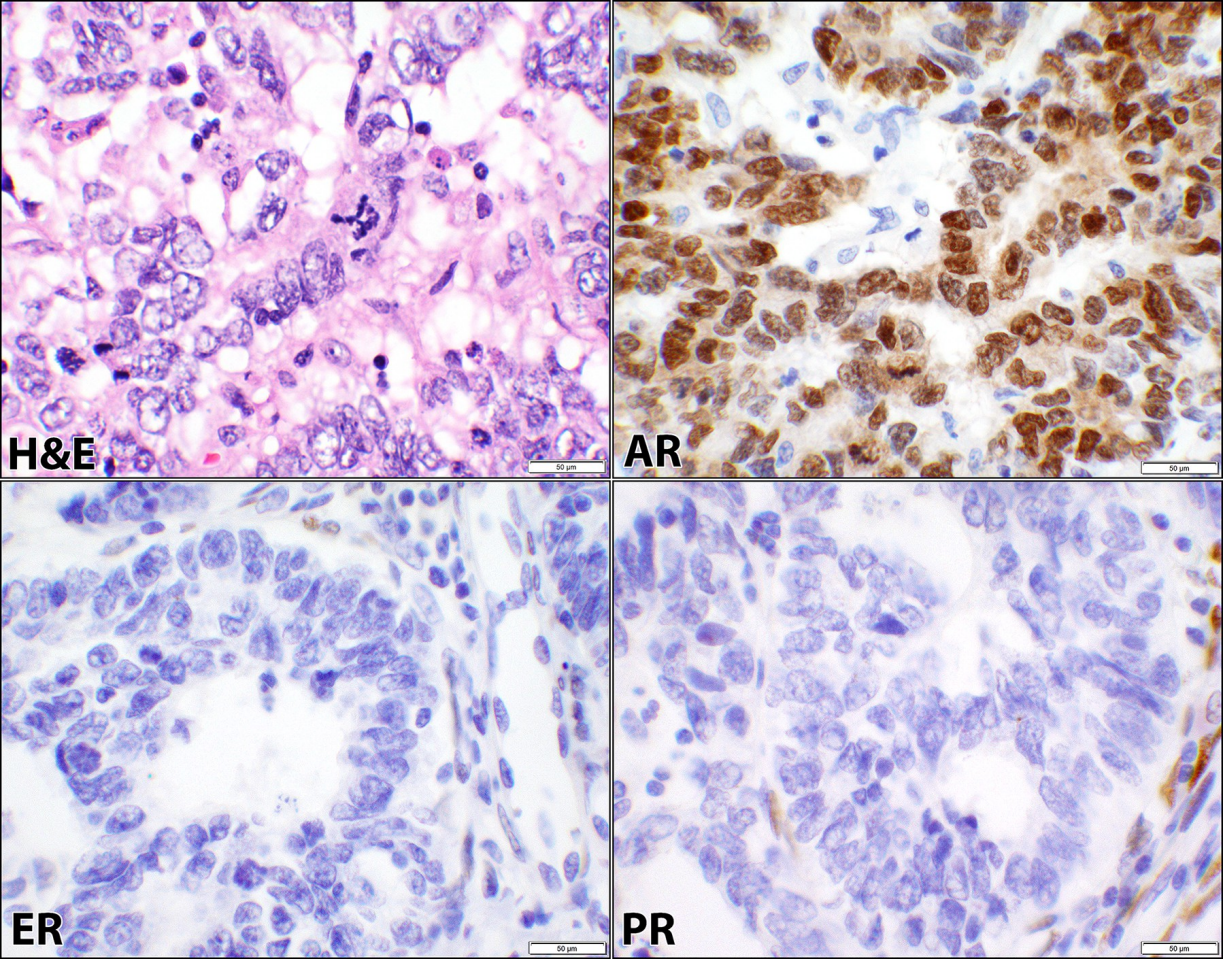
Androgen, estrogen, and progesterone receptors in serous carcinoma. Hematoxylin and eosin (H&E) stain of a case of uterine serous carcinoma showing papillary and glandular patterns where cells exhibit high-grade cytology, mitotic figure, and marked nuclear pleomorphism (case #40, S1 Table). The corresponding androgen receptor (AR), estrogen receptor (ER), and progesterone receptor (PR) immunostains show positive nuclear staining with AR and negative nuclear staining of the malignant cells for ER and PR (40× objective).

**Fig 3:**
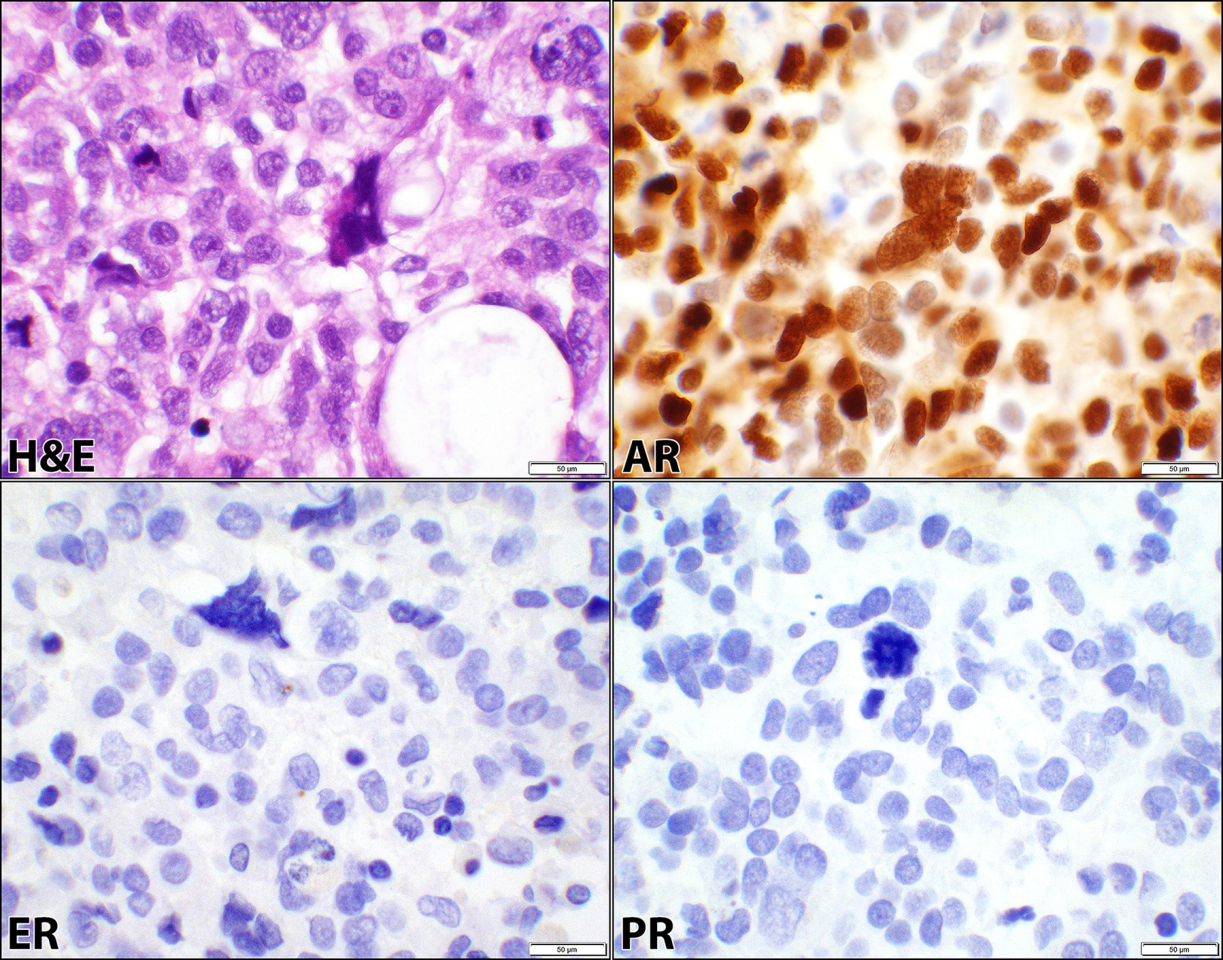
Androgen, estrogen, and progesterone receptors in clear cell carcinoma. Hematoxylin and eosin (H&E) stain of a case of clear cell carcinoma of the endometrium showing tubulocystic and solid architectural patterns, variably pleomorphic and cuboidal cells, and clear cytoplasm (case #53, S1 Table). The corresponding androgen receptor (AR), estrogen receptor (ER), and progesterone receptor (PR) immunostains show positive nuclear staining for AR and negative staining of the malignant cells for ER and PR (40× objective).

**Fig 4:**
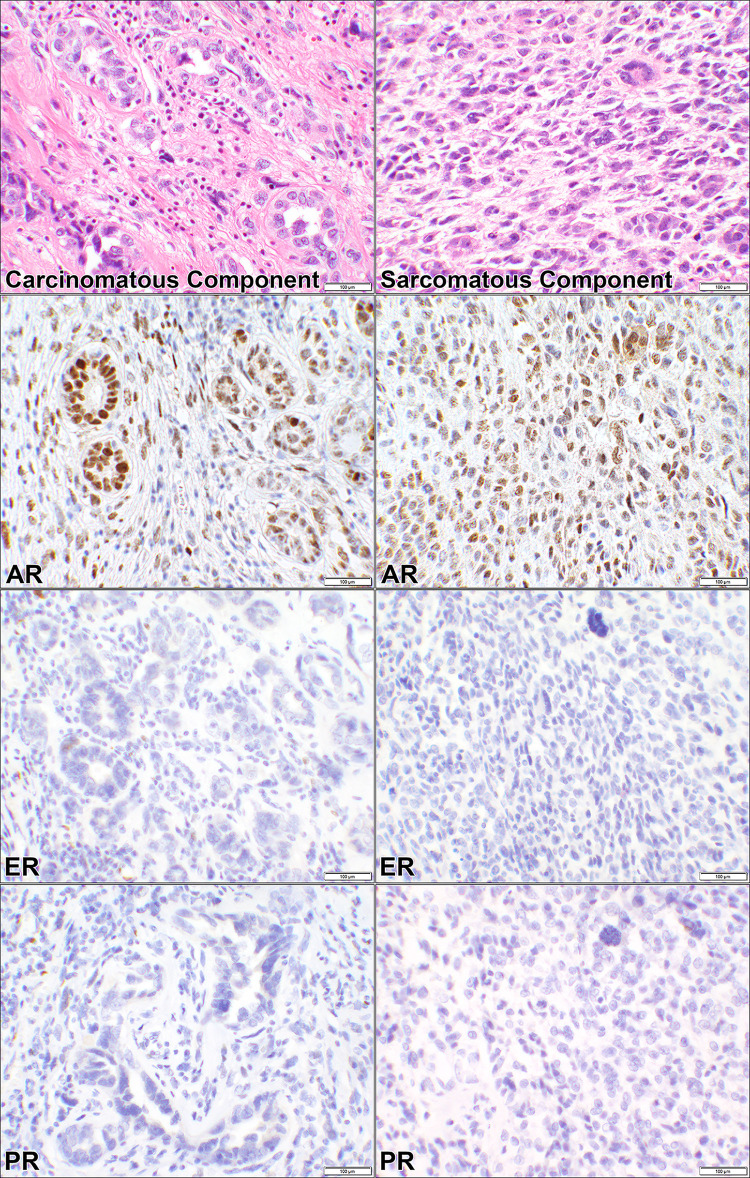
Androgen, estrogen, and progesterone receptors in carcinosarcoma. Hematoxylin and eosin (H&E) stain of a case of uterine carcinosarcoma showing a biphasic pattern composed of high-grade carcinomatous and sarcomatous components (case #60, S1 Table). The corresponding androgen receptor (AR), estrogen receptor (ER), and progesterone receptor (PR) immunostains show positive nuclear staining for AR in both carcinomatous and sarcomatous components. ER and PR are negative in the two components of the cancer (20× objective).

The cases were comprised of SCA (13 patients), CCCA (6 patients), CS (6 patients), DECA (5 patients), and MNCA (1 patient) as listed in Tables 1–4. Since these cancers were high-grade, FIGO gradings were omitted based on the WHO recommendations (S1 Table). ER was positive in 48% (15/31), PR in 35% (11/31), and AR in 68% (21/31) of the cancers in this category as shown in Table 1 and represented in Figs 2–4 for SCA, CCCA, and CS respectively. Carcinomatous and sarcomatous components were both positive for AR in carcinosarcomas (Fig 4). Overall, the cellular positivity ranged from 3% to 95% with combined 2+ & 3+ intensities in 35% (11/31), 32% (10/31), and 65% (20/31) of ER, PR, and AR respectively (S1 Table), indicative of higher rate and stronger expression of AR in this diagnostic cancer category. Fisher’s Exact test had returned insignificant *p*-values (0.4 and 0.2) when ER was compared to PR and AR stains (Table 1). However, there was a significant difference (*p*-value = 0.02) when the comparison was made between AR and PR (Table 1).

MMR stain reactions showed intact proteins in 23 cases where loss of MLH1 and PMS2 were observed in 3, all in DECAs, and the tests were not performed for 5 patients (S1 Table). MMR was significantly different (*p*-value ≤ 0.03) when compared to each receptor except for PR (*p*-values of 0.3) using Fisher’s Exact test (Table 2). The receptor expressions were significantly different from p53 with *p*-values of ≤ 0.01 obtained from Fisher’s Exact test (Table 3). When the receptors were compared to the BMI (≥ 25), the results were variable. AR versus BMI showed no significant difference (*p*-value = 0.4). ER and PR, on the other hand, were significantly different (*p*-values ≤ 0.2) meaning a concordance between BMI and AR (Table 4).

Most notably, all 6 (100%) cases of CCCA were negative for ER and PR staining whereas 50% of them had expressed AR. Also, one of the SCA cases while being positive for AR was negative for ER and PR (case # 40; S1 Table). Similarly, AR was expressed in 100% (6/6) of cases with CS but had ER and PR expression in 67% and 50% of the cancers respectively. Six cases of DECA and MNCA were negative for all the three receptors.

### Met (metastatic carcinoma)

There were four cases in this category. Patients’ ages ranged between 60 to 70 years with a median of 66.5. All patients were postmenopausal. All four patients had received chemotherapy, two of whom had also been treated with an anti-estrogen agent (anastrozole). Only one case in this group had her primary carcinoma excised in our institution (case #71, S1 Table) which her primary surgery had been 2 years prior (case #26, S1 Table). The other three cases had their original cancers resected in outside institutions with uncertain tumor type to us but appeared to be EEC. All four carcinomas were positive for the three receptors except for one in which PR was negative. Overall cellular positivity ranged from 10% to 95% with the intensities of 2+ and 3+.

MMR stains showed intact protein expressions in two cases and loss of MLH1 and PMS2 were seen in another two samples (S1 Table). p53 was performed on only one patient. This diagnostic category is very limited by the very low number of cases. Although for all comparisons, including BMIs, the obtainable *p*-values did indicate significant differences (Tables 1–4).

## Discussion

This study shows strong expression of AR in 85% of all endometrial malignancies which postures as a potential targeted therapy which also has been suggested by other investigators [4, 14, 23]. AR, ER, and PR expressions are seen in a high number of endometrioid and metastatic carcinomas (97%-100%) and their positivity are very similar (Table 1). Most notable findings are among the NEEC where the receptors’ expressions are variable or even absent (total absence in DECA). For example, AR has been expressed in 50% of clear cell carcinomas with no co-expression of either ER or PR. Likewise, AR expression is seen in 100% of carcinosarcomas while ER and PR are co-expressed in 67% and 50% of the cancers respectively (Table 1). Another example is expression of AR in 92% of the serous carcinomas but the ER and PR counterparts are in 85% and 62% (Table 1). Interestingly, AR positivity has been seen in a single case despite the lack of ER and PR expressions (case# 40, S1 Table). Generally, if expressed, either AR is the sole expressed receptor, or the rate of its expression is higher than ER and PR (Table 1). Tangen et al. have reported that AR is more often expressed in metastatic lesions than their respective primary cancers. Also, in their study, 39% of the metastatic endometrial cancers have shown a higher rate of AR expression than ER [14]. In our series, however, we could not compare the hormone receptors’ expression of the primary cancers with their respective metastatic lesions since we had only one case with known primary diagnosis: posing as a limitation in the metastatic diagnostic category.

In order to compare our findings with those in the literature, we identified three publications in which the hormonal receptors are classified in each diagnostic category [4, 14, 23]. In one of the three reports, 718 biopsies from primary endometrial cancers and 298 metastatic lesions from 142 patients have been studied [14]. While they have specifically reported the expression of AR in different types of uterine malignancies, it is difficult to accurately extract and correlate the data to ER and PR for the same types of cancers. Therefore, their findings regarding ER and PR could not be included in Table 5. However, they have noted that 67% of the EECs have expressed AR [14]. Based on the tabulated three studies and the current series, AR is expressed in 64% of all reported endometrial malignancies as opposed to the findings in our study alone where 85% of the cancers have AR expression (Table 5). The differences of AR expression in our series versus the other studies may be related to the sample size of our series, although two of the studies have had smaller case numbers than ours. In fact, all cited works in Table 5 have limitations and/or biases. Our series is biased because of the preselection of 71/937 cases based on the ER and PR tests performed non-randomly during the course of patient care. Cao et al. ‘s study is skewed because of the focus on a higher number of DECA lesions [23]. Zadeh et al. have used tissue micro-array (TMA) on selected cases where basis for the case selection has not been described [4]. Tangen et al. [14] also have used a TMA which its construction has been referenced to a publication of 10 years earlier [24], where the basis for the case selection has not been elaborated in either paper. These biases/limitations may have contributed to the variability of the three receptors expression rates in the four studies (Table 5). Regardless of the limitations, AR expression stands out at high rates in these studies even more than ER and PR in most instances (Table 5).

**Table 5:** Summary of literature review delineating expression of the hormonal receptors in the endometrial malignancies.

Authors	Institution	Year	EEC				SCA				CCCA				CS			
** **				**AR**	**ER**	**PR**	** **	**AR**	**ER**	**PR**	** **	**AR**	**ER**	**PR**	** **	**AR**	**ER**	**PR**
** **			**Tot**	**+n** (**+%**)	**+n** (**+%**)	**+n** (**+%**)	**Tot**	**+n** (**+%**)	**+n** (**+%**)	+n (**+%**)	**Tot**	**+n** (**+%**)	**+n** (**+%**)	**+n** (**+%**)	**Tot**	**+n** (**+%**)	**+n** (**+%**)	**+n** (**+%**)
Current series	UCLA	2023	36	35 (**97**)	35 (**97**)	35 (**97**)	13	12 (**92**)	11 (**85**)	8 (**62**)	6	3 (**50**)	0 (**0**)	0 (**0**)	6	6 (**100**)	4 (**67**)	3 (**50**)
Cao et al. [23]	HNSM	2021	9	6 (**67**)	6 (**67**)	2 (**20**)	8	7 (**88**)	4 (**50**)	4 (**50**)	12	1 (**9**)	1 (**9**)	1 (**9**)	10	8 (**80**)	8 (**80**)	6 (**60**)
Zadeh et al. [4]	UVC	2018	20	11 (**65**)	18 (**90**)	17 (**85**)	10	7 (**70**)	8 (**80**)	5 (**50**)	10	3 (**30**)	6 (**60**)	1 (**10**)	10	5 (**50**)	4 (**40**)	3 (**30**)
Tangen et al. [14]	UIB	2016	574	386 (**67**)	LP	LP	65	36 (**55**)	LP	LP	28	7 (**25**)	LP	LP	32	13 (**41**)	LP	LP
			**DECA**				**MNCA**				**Met**				**All**			
				**AR**	**ER**	**PR**		**AR**	**ER**	**PR**		**AR**	**ER**	**PR**		**AR**	**ER**	**PR**
			**Tot**	**+n** (**+%**)	**+n** (**+%**)	**+n** (**+%**)	**Tot**	**+n** (**+%**)	**+n** (**+%**)	**+n** (**+%**)	**Tot**	**+n** (**+%**)	**+n** (**+%**)	**+n** (**+%**)	**Tot**	**+n** (**+%**)	**+n** (**+%**)	**+n** (**+%**)
Current series	UCLA	2023	5	0 (**0**)	0 (**0**)	0 (**0**)	1	0 (**0**)	0 (**0**)	0 (**0**)	4	4 (**100**)	4 (**100**)	3 (**75**)	71	60 (**85**)	54 (**76**)	49 (**69**)
Cao et al. [23]	HNSM	2021	16	10 (**63**)	NP	NP		NR	NR	NR		NR	NR	NR	55	32 (**58**)	19 (**35**)	13 (**24**)
Zadeh et al. [4]	UVC	2018		NR	NR	NR		NR	NR	NR		NR	NR	NR	50	26 (**52**)	36 (**72**)	26 (**52**)
Tangen et al. [14]	UIB	2016	13	1 (**7**)	LP	LP		0 (**0**)	LP	LP	Unclear data				712	442 (**62**)	LP	LP
														**Total**	888	569 (**64**)	109 (**72**)	88 (**50**)

**EEC**, endometrial endometrioid cancer; **SCA**, serous carcinoma; **CCCA**, clear cell carcinoma; **CS**, carcinosarcoma; **DECA**, dedifferentiated/undifferentiated endometrial carcinoma; **MNCA**, mesonephric like carcinoma; **Met**, metastatic carcinoma; **Tot**, total cases; **AR**, androgen receptor; **ER**, estrogen receptor; **PR**, progesterone receptor; **+n**, number of positives; **+%**, percentage of positives; **NP**, Data Not provided; **NR**, not reported; **UCLA**, University of California, Los Angeles; **HNSM**; Donald and Barbara Zucker School of Medicine at Hofstra/Northwell School of Medicine, Hempstead, New York; **UVC**, University of Virginia, Charlottesville; **UIB**, University of Bergen, Norway; **LP**, ER & PR data were lumped together, therefore not represented in this table.

Hormonal therapy targeting both ER and PR is effective in treatment of endometrial cancers when the receptors are expressed [15, 25]. Overall, since metastatic-endometrial carcinomas and NEEC express AR, this may serve as a potential therapeutic target for these patients based on the historical targeted treatment of male prostate and female breast cancers with anti-androgen/AR agents [6–9]. Targeting AR has not been explored extensively in this disease model which could be a potential alternative hormonal anticancer therapy for ECs using androgen and/or AR antagonists [26]. Despite antiproliferative effects of androgen in normal endometrium, recently androgen antagonists have been used as a therapeutic agent in endometrial cancers in the mouse model [27]. In a recent meeting of the Society of Gynecology Oncology (SGO), Westin et al. presented their clinical trial study regarding phase II trial with safety lead of enzalutamide in combination with paclitaxel and carboplatin for advanced or recurrent endometrioid carcinomas. They have reported the combination of enzalutamide, carboplatin, and paclitaxel was tolerated by 81 patients showing promising clinical outcomes in chemo-naive, advanced, or recurrent endometrioid cancers [28].

Enzalutamide, an androgen antagonist, is an FDA approved agent for use in prevention of the androgen dependent growth of prostate cancers [29]. This drug prevents the nuclear translocation of AR and its sequence-specific binding to DNA, resulting in cellular apoptosis in endometrial cancers [27].

Phosphatase and tensin homolog (PTEN) loss is a frequent genetic finding in both prostatic and type 1 endometrial cancers that results in activation of AKT signaling [30, 31]. Koivisto et al., in mouse models of prostate cancer, have shown oral administration of enzalutamide results in a reduction of the tumor growth. They have seen similar effects in endometrial cancers [27]. Short-term use of enzalutamide, as a single-agent, resulted in increased apoptosis and decreased tumor burden in the endometrial cancers after 8-weeks of treatment. However, prolonged use of enzalutamide failed to sustain tumor reduction and resulted in overexpression of p53 protein. Subsequently, resistance to enzalutamide was seen in mouse models with low-grade ECs [27]. They also have demonstrated that co-administration of progesterone and enzalutamide may produce synergistic effects by blocking epithelial proliferation through stromal PR signaling and inducing epithelial apoptosis through enzalutamide [27]. Manning-Geist et al. have also suggested the role of enzalutamide as a potential treatment option in selected patients with AR positive high-grade and low-grade serous ovarian cancers [32]. Due to lack of the long term anti-endometrial cancer therapeutic effects of enzalutamide, combination therapies targeting more than one hormone signaling axis may be necessary in future studies. To our knowledge, there are no clinical trials underway using androgen/androgen-receptor targeted therapy in human endometrial cancers at this time, except for preclinical mouse models [27] and a preliminary human study [28].

Based on our study, there is a lack of concordance between MMR and p53, with the three receptors. MMR’s losses’, which mainly has occurred in EECs, were in a small number (17%) of cases as opposed to 85% of AR expression in this series. Although, BMI of ≥25 did correlate with the three receptors, there was an exception. In NEEC, AR did correlate with the BMI where there was no significant difference (*p*-value = 0.4) by the Fisher’s exact test (Table 4). Cao et al. have reported that free testosterone and DHEA-S levels are increased in late postmenopausal women which correlate with abdominal fat accumulation [13]. In this series, 96% (68/71) of the patients were postmenopausal and 76% (54/71) were overweight or obese. This study is limited by its small sample size. Further studies are warranted to explore the relationship between AR and the other markers in a randomized uniform patient population.

## Conclusions

Our findings show that androgen receptor is strongly expressed, more so than ER and PR, in most endometrial cancers, notably in the high-grade malignancies. Also, our findings confirm the results of the recent studies that found a significant expression of AR in high-grade serous and clear cell carcinomas in the absence of ER and PR positivity. These findings provide support for evaluating AR expression in endometrial malignancies, particularly in the specific subtypes where ER and PR are not expressed. Since there is no evidence of adhering to the REMARK guidelines [22] in the published reports [4, 14, 23], larger studies may be warranted to further evaluate the potential role of AR for enzalutamide therapy in ECs, especially in high grade cancers which only express this receptor.

## Supporting information

S1 Table Summary of the patients’ data. (PDF)
