# Supplementary material for: Androgen receptor and its correlation with estrogen and progesterone receptors, aimed for identification of cases for future anti-androgen therapy in endometrial cancers
Source: PLoS One. 2023 Sep 19;18(9):e0291361. doi: 10.1371/journal.pone.0291361 (PMC10508627; doi:10.1371/journal.pone.0291361)
Supplement: S1 Table — (PDF) [file pone.0291361.s001.pdf]

S1 Table. Summary of the demographic information, diagnostic characteristics, and immunohistochemical reactions of the three receptors plus MMR, p53, BMI values, and months of survival after the initial diagnosis in the patients with endometrial cancers

No.	Age	Diagnosis	FIGO	ER		PR		AR		MMR	p53	BMI	M-Alive	Rx
				Cells %	Intensity	Cells %	Intensity	Cells %	Intensity					
1	70	EEC	1	80%	3+	40%	2+	50%	2+	Intact	WT	29.19	77 §	No
2	68	EEC	1	60%	3+	80%	3+	70%	3+	Intact	WT	31.37	75 §	No
3	80	EEC	1	30%	1+	50%	2+	60%	2+	Loss1	AE	23.08	75 §	No
4	69	EEC	1	70%	3+	70%	2+	90%	3+	Intact	AE	31.09	74 §	No
5	67	EEC	1	60%	1+	80%	3+	60%	2+	Loss1	NP	45.00	71 §	No
6	54	EEC	1	80%	2+	80%	3+	70%	2+	Intact	NP	34.54	49 §	No
7	65	EEC	1	70%	2+	90%	2+	90%	2+	Intact	WT	33.66	49 §	No
8	88	EEC	1	70%	2+	40%	3+	90%	3+	Intact	WT	21.05	34 §	Ch
9	74	EEC	1	90%	3+	90%	3+	40%	3+	Intact	WT	24.87	27 §	No
10	60	EEC	1	60%	2+	60%	2+	60%	2+	Intact	NP	46.27	24 §	No
11	64	EEC	1	Neg		Neg		Neg		Loss1	WT	26.67	22 §	No
12	76	EEC	1	85%	3+	80%	3+	80%	3+	Intact	NP	19.61	21 §	No
13	62	EEC	1	30%	3+	30%	3+	30%	3+	Intact	WT	22.72	21 §	No
14	67	EEC	1	90%	3+	90%	3+	90%	3+	Intact	NP	34.09	20 §	No
15	57	EEC	1	65%	2+	70%	3+	10%	2+	Intact	NP	46.99	19 §	No
16	65	EEC	1	65%	3+	60%	3+	70%	3+	Intact	WT	42.84	17 §	No
17	62	EEC	1	80%	2+	80%	2+	70%	2+	Intact	WT	45.16	17 §	No
18	65	EEC	1	70%	2+	40%	2+	70%	2+	Intact	NP	24.60	16 §	No
19	68	EEC	1	90%	3+	90%	3+	90%	3+	Intact	NP	43.64	15 §	No
20	41	EEC	1	90%	3+	70%	3+	60%	3+	Intact	NP	29.29	12 §	No
21	88	EEC	2	40%	1+	10%	1+	10%	1+	Intact	NP	36.57	30	No
22	53	EEC	2	30%	2+	50%	2+	50%	2+	Intact	AE	22.53	74 §	No
23	57	EEC	2	70%	3+	70%	2+	20%	2+	Intact	NP	34.07	57 §	No
24	59	EEC	2	40%	2+	80%	3+	40%	2+	Intact	WT	42.57	46 §	No
25	59	EEC	2	50%	3+	80%	3+	50%	3+	Loss2	WT	27.50	40 §	No
26*	61	EEC	2	85%	3+	65%	3+	80%	3+	Intact	WT	27.81	38 §	Ch+Hr
27	61	EEC	2	10%	3+	10%	3+	10%	3+	Intact	NP	38.96	37 §	No
28	69	EEC	2	70%	2+	70%	2+	70%	2+	Intact	WT	19.67	29 §	Ch
29	70	EEC	2	80%	3+	40%	3+	40%	3+	Intact	WT	21.51	24 §	No
30	69	EEC	2	90%	3+	90%	3+	90%	3+	Intact	WT	36.18	24 §	No
31	50	EEC	2	90%	3+	90%	3+	90%	3+	Loss2	WT	27.56	21 §	No
32	58	EEC	2	30%	2+	2%	2+	70%	2+	Intact	WT	29.49	16 §	No
33	42	EEC	2	90%	3+	90%	3+	50%	2+	Intact	WT	26.35	13 §	No
34	84	EEC	3	10%	2+	10%	2+	80%	2+	Intact	WT	19.57	29 §	Ch
35	75	EEC	3	30%	1+	5%	1+	10%	3+	Loss1	WT	14.27	13	Ch
36	59	EEC	3	50%	2+	30%	2+	60%	2+	Intact	WT	29.61	14 §	Ch
37	78	SCA		Neg		Neg		Neg		Intact	AE	26.35	34	Ch
38	67	SCA		10%	2+	20%	3+	90%	3+	NP	AE	67.67	67 §	Ch
39	78	SCA		70%	1+	Neg		70%	3+	Intact	AE	33.44	67 §	NF
40	71	SCA		Neg		Neg		90%	3+	Intact	AE	33.82	63 §	Ch
41	70	SCA		40%	3+	5%	3+	5%	3+	Intact	AE	32.01	54 §	Ch
42	76	SCA		95%	3+	25%	2+	95%	3+	Intact	AE	29.51	51 §	Ch
43	60	SCA		80%	3+	75%	3+	90%	3+	Intact	AE	32.19	36 §	Ch
44	70	SCA		95%	3+	95%	3+	40%	3+	Intact	AE	30.91	31 §	Ch
45	76	SCA		90%	3+	10%	2+	90%	3+	NP	AE	25.08	3	No
46	69	SCA		20%	1+	15%	1+	90%	3+	NP	AE	35.43	21 §	Ch
47	61	SCA		20%	3+	Neg		90%	3+	Intact	AE	45.00	17 §	Ch
48	68	SCA		25%	3+	5%	2+	25%	3+	Intact	AE	18.85	14 §	Ch
49	61	SCA		35%	1+	Neg		95%	3+	Intact	AE	45.00	12 §	Ch
50	45	CCCA		Neg		Neg		Neg		NP	NP	22.75	75 §	Ch
51	78	CCCA		Neg		Neg		5%	3+	Intact	AE	40.35	6	Ch
52	67	CCCA		Neg		Neg		5%	3+	Intact	AE	37.11	16 §	Ch
53	67	CCCA		Neg		Neg		50%	3+	Intact	AE	26.78	14 §	Ch
54	58	CCCA		Neg		Neg		Neg		Intact	AE	25.79	13 §	Ch
55	64	CCCA		Neg		Neg		Neg		Intact	AE	26.18	11 §	Ch
56	73	CS		10%	2+	10%	2+	10%	2+	NP	AE	27.57	13	Ch
57	82	CS		Neg		Neg		10%	1+	Intact	AE	29.84	30	Ch
58	81	CS		3%	2+	Neg		80%	3+	Intact	NP	31.46	1	No
59	65	CS		40%	3+	10%	2+	40%	3+	Intact	NP	24.89	27 §	Ch
60	57	CS		Neg		Neg		80%	2+	Intact	AE	25.40	2	No
61	63	CS		30%	1+	25%	2+	70%	2+	Intact	AE	22.62	13	Ch
62	79	DECA		Neg		Neg		Neg		Intact	AE	21.16	76 §	Ch
63	54	DECA		Neg		Neg		Neg		Loss1	AE	25.28	4	Ch
64	68	DECA		Neg		Neg		Neg		Loss1	AE	33.28	35 §	Ch
65	53	DECA		Neg		Neg		Neg		Loss1	AE	30.09	33 §	Ch
66	62	DECA		Neg		Neg		Neg		Intact	AE	32.28	20 §	Ch
67	83	MNCA		Neg		Neg		Neg		Intact	WT	20.85	12	Ch
68	60	Met	1	90%	3+	90%	3+	60%	3+	Intact	NP	37.08	43 §	Ch+Hr
69	70	Met	2	80%	3+	60%	3+	90%	3+	Loss1	NP	44.34	33 §	Ch
70	70	Met	2	70%	3+	90%	3+	10%	2+	Loss1	NP	44.34	33 §	Ch
71*	63	Met	2	95%	3+	Neg		95%	3+	Intact	WT	25.74	18 §	Ch+Hr

ER, estrogen receptor; **PR**, progesterone receptor; **AR**, androgen receptor, **EEC**, endometrial endometrioid cancer; **SCA**, serous carcinoma; **CCCA**, clear cell carcinoma; **CS**, carcinosarcoma; **DECA**, dedifferentiated/undifferentiated endometrial carcinoma; **MNCA**, mesonephric like carcinoma; **Met**, metastatic carcinoma; **MMR**, mismatch repair; **Loss1**, loss of MLH1 & PMS2; **Loss2**, loss of MSH2 & MSH6; **WT**, wild type; **AE**, aberrant expression; **NP**, not performed; **BMI**, body mass index (kg/m^2), **Red,** obese (BMI ≥30), **Orange,** overweight (BMI ≥25 to <30), **Blue,** underweight (BMI <18.5); **M-Alive**, months alive (in **Red,** expired), **§** = still alive as of 2023-05-13; **Rx**, therapy; **Ch**, chemotherapy; **Hr**, hormonal therapy; **NF**, no follow-up; Blue highlight, patient was menstrual; *, same patient.
